# Convergent evolution in nucleocapsid facilitated SARS-CoV-2 adaptation for human infection

**DOI:** 10.1128/jvi.02091-24

**Published:** 2025-06-12

**Authors:** Kumari G. Lokugamage, Yiyang Zhou, R. Elias Alvarado, Jessica A. Plante, Yani Ahearn, Jennifer Chen, Leah Estes, William Meyers, Jakob Nilsson, Andrew L. Routh, David H. Walker, Vineet D. Menachery, Bryan A. Johnson

**Affiliations:** 1 Department of Microbiology and Immunology, The University of Texas Medical Branch 547647, Galveston, Texas, USA; 2 Department of Biochemistry and Molecular Biology, The University of Texas Medical Branch 198643, Galveston, Texas, USA; 3 World Reference Center of Emerging Viruses and Arboviruses, The University of Texas Medical Branch 12338, Galveston, Texas, USA; 4 Department of Pathology, The University of Texas Medical Branch 198642, Galveston, Texas, USA; 5 Novo Nordisk Foundation Center for Protein Research, Faculty of Health and Medical Sciences, University of Copenhagen 4321, Copenhagen, Denmark; 6 Department of Immunology and Microbiology, Scripps Research, La Jolla, California, USA; 7 Center for Biodefense and Emerging Infectious Diseases, The University of Texas Medical Branch 12338, Galveston, Texas, USA; 8 Department of Pediatrics & Emory Vaccine Center, Emory University 1371, Atlanta, Georgia, USA; 9 Institute for Human Infection and Immunity, The University of Texas Medical Branch 551582, Galveston, Texas, USA; 10 Center for Tropical Diseases, The University of Texas Medical Branch 12338, Galveston, Texas, USA; Cornell University Baker Institute for Animal Health, Ithaca, New York, USA

**Keywords:** SARS-CoV-2, COVID-19, nucleocapsid, variants of concern

## Abstract

During the early stages of the COVID-19 pandemic, several SARS-CoV-2 variants of concern (VOCs) independently acquired mutations in the highly variable 203–205 amino acid region of the nucleocapsid (N) protein including R203K + G204R (found in the Alpha, Gamma, and Omicron variants), R203M (in Delta), and T205I (in Beta). In previous research, we demonstrated that the R203K + G204R mutation significantly enhances SARS-CoV-2 N phosphorylation, which subsequently increases viral fitness and pathogenesis. In this study, we investigated the effects of the R203M and T205I mutations on SARS-CoV-2 infection. Using reverse genetics, we introduced these mutations into the early pandemic Washington-1 (WA-1) strain and observed that both the R203M and T205I mutants enhanced replication and viral fitness. However, unlike the R203K + G204R mutant, the R203M and T205I mutants caused only moderate changes in lung pathology. Notably, each mutation—R203K + G204R, R203M, and T205I—induced distinct patterns of N phosphorylation, which likely contribute to the observed phenotypic differences between the mutants. Interestingly, when bat cells expressing human ACE2 were infected with these mutants, we observed a reduction, rather than an enhancement, in both SARS-CoV-2 replication and N phosphorylation. Collectively, our findings suggest that the R203K + G204R, R203M, and T205I mutations are a result of convergent evolution and reflect how SARS-CoV-2 has adapted for human infection.

**IMPORTANCE:**

After its emergence, SARS-CoV-2 rapidly adapted to human infection, acquiring numerous mutations across its genome. Many of these mutations remain uncharacterized. This study examines a mutational hotspot among SARS-CoV-2 variants: residues 203–205 of the nucleocapsid (N) protein. We demonstrate that three unique mutations identified in this region among variants of concern enhance infection in human cells and animal models while eliciting distinct patterns of N protein phosphorylation. Intriguingly, these same mutations reduce both N protein phosphorylation and viral replication in bat cells. These findings suggest that each mutation represents independent adaptation by variants of concern for human infection. Importantly, this study underscores the critical role of these mutations in facilitating the expansion of SARS-CoV-2 into human populations and highlights the potential for similar mutations to drive future zoonotic coronavirus outbreaks.

## INTRODUCTION

Since its emergence in December 2019, SARS-CoV-2 has continued to evolve, resulting in genetic variants with distinct mutational profiles. Among the many variants identified, five (Alpha, Beta, Delta, Gamma, and Omicron) have been classified by the World Health Organization (WHO) as variants of concern (VOC) due to their potential for enhanced transmission, pathogenicity, or immune escape (1). Characterization of VOCs has predominantly focused on mutations within the spike (S) gene, given its central role in transmission and vaccine-induced immunity (2). However, each VOC contains mutations throughout the SARS-CoV-2 genome (2), which have the potential to impact viral fitness or pathogenesis.

For example, all five VOCs harbor one of three mutations in the 203–205 region of the nucleocapsid (N) gene. These include the R203K and G204R (KR) mutations in Alpha, Gamma, and Omicron, the R203M mutation in Delta, and the T205I mutation in Beta (2). Previous work by our lab and others demonstrated that one of these mutations, the KR mutant, is sufficient to enhance SARS-CoV-2 infection (3–5). Using a reverse genetic system to introduce the mutation into the early pandemic Washington-1 (WA-1) background, the KR mutant was sufficient to enhance replication in cell culture and pathogenesis in the golden Syrian hamster model (3, 5). While other mechanisms have been suggested (6, 7), our lab demonstrated that the KR mutation altered the phosphorylation of the serine arginine (SR) domain of SARS-CoV-2 N (3). Because the phosphorylation of the SR domain is hypothesized to regulate N protein function (8–10), these data suggest that the KR mutation may represent a fine-tuning of SARS-CoV-2 N protein function for human infection through the regulation of its phosphorylation state. Whether the R203M and T205I mutations elicit similar effects remains unclear.

Here, we characterize the effects of the R203M and T205I mutations on SARS-CoV-2 infection. By introducing these mutations into the WA-1 background using a reverse genetic system (11), we demonstrate that, like KR, the R203M and T205I mutations are both sufficient to enhance replication and fitness compared to wild-type (WT) SARS-CoV-2 *in vitro*. Surprisingly, when the relative fitness between the SARS-CoV-2 N mutants was examined by direct competition in human respiratory cells, the T205I mutation, rather than the KR mutation (extant in nearly all currently circulating SARS-CoV-2 lineages), was found to have the highest replication fitness. During *in vivo* infection of golden Syrian hamsters, the R203M and T205I mutations enhanced viral titer and lung pathology without affecting weight loss. Examining SARS-CoV-2 N phosphorylation, we find that the KR, R203M, and T205I mutations each confer a different phosphorylation pattern, accounting for their differential impact on infection. Finally, by infecting cells derived from *Eptesicus fuscus* (big brown bats) expressing human ACE2 (12), all three SARS-CoV-2 N mutations exhibited reduced replication and N phosphorylation relative to SARS-CoV-2 WT. Together, these data suggest that the KR, R203M, and T205I mutations are the result of convergent evolution modifying the SARS-CoV-2 N protein for enhanced human infection.

## RESULTS

### Variant mutations in the SR domain of nucleocapsid enhance replication

**Fig 1:**
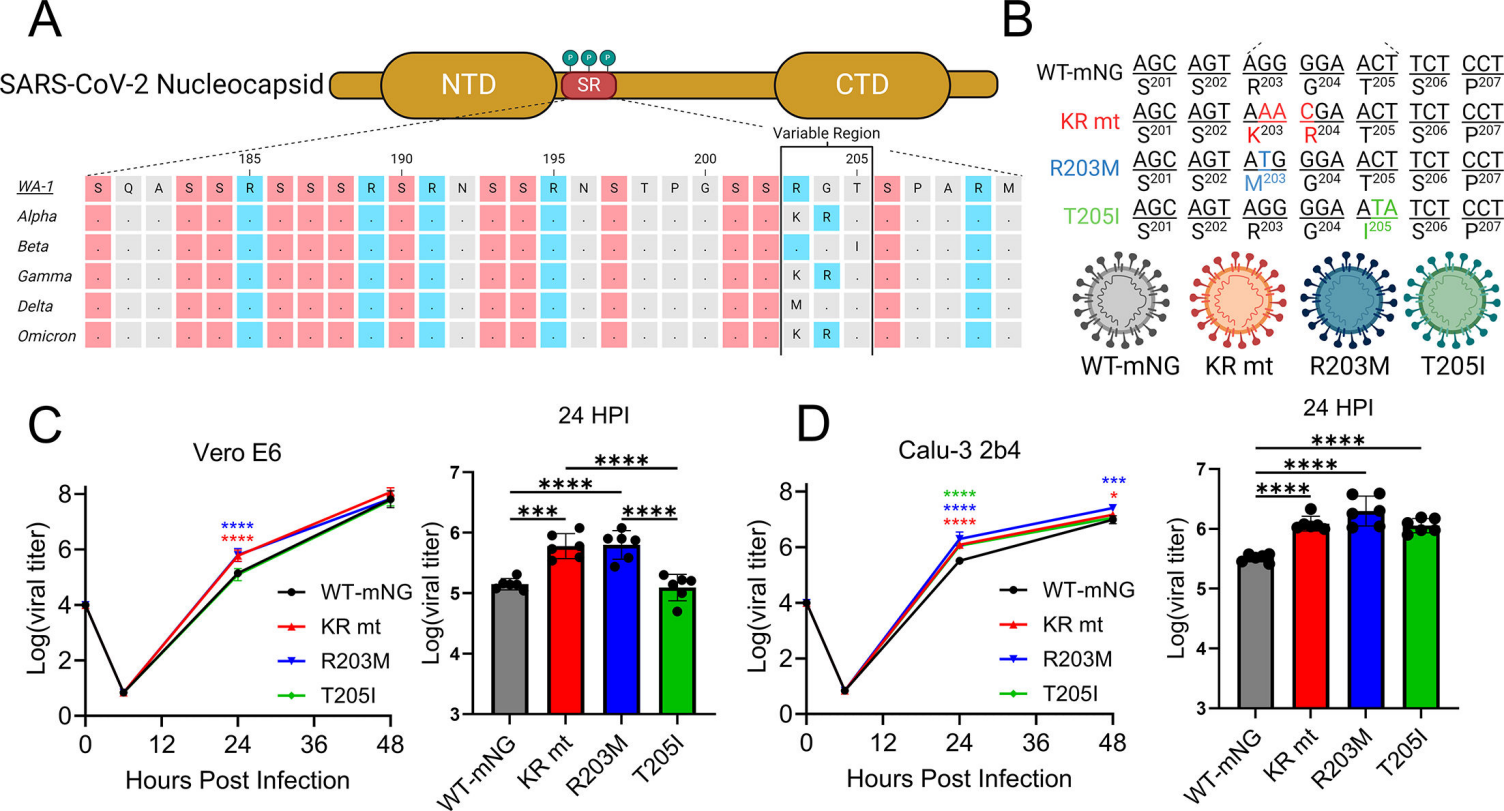
The KR, R203M, and T205I mutations enhance SARS-CoV-2 replication *in vitro.* (**A**) Schematic detailing the serine-arginine (SR) domain of each variant of concern. Serine residues are colored in red, arginine residues in blue. (**B**) Schematic of the KR, R203M, and T205I mutations being introduced into the Washington-1 (WA-1) strain. (**C, D**) Viral titers from Vero E6 (**C**) or Calu-3 2b4 (**D**) cells infected with WT-mNG (black) or the KR (red), R203M (blue), or T205I (green) mutants at an MOI of 0.01. Full growth curves are shown on the left side of each panel, while bar graphs show mean viral titer at 24 hpi. (*n* = 6). Error bars are ± s.d., with individual replicates displayed as dots on bar graphs. Statistical significance was determined by one-way ANOVA followed by Tukey’s multiple comparison test. (*) *P* ≤ 0.05; (***) *P* ≤ 0.001; (****) *P* ≤ 0.0001.

Each variant of concern (VOC) harbors one of three possible mutations between residues 203–205 of the serine-arginine (SR) domain of SARS-CoV-2 nucleocapsid (N), including the R203K + G204R (KR), R203M, and T205I mutations, hereafter collectively referred to as the SR mutations (Fig. 1A) (2). Using a reverse genetic system based on the Washington-1 (WA-1) background, we previously generated a recombinant SARS-CoV-2 harboring the KR mutation, demonstrating that this mutation was sufficient to augment SARS-CoV-2 fitness and pathogenesis (3). While we also generated a recombinant virus harboring the R203M mutation, we performed only a limited characterization (3). Thus, it is unclear if the R203M and T205I mutations elicit equivalent changes in SARS-CoV-2 infection.

To determine whether the R203M and T205I mutants phenocopy the KR mutant, recombinant SARS-CoV-2 WA-1 harboring the T205I mutation was generated by reverse genetics (Fig. 1B) (11). All mutant viruses were generated in a background where ORF7 is replaced by a mNeonGreen reporter, which attenuates pathogenesis *in vivo* (3). After recovery of the recombinant T205I virus, the replication of all three SR mutants was assessed relative to SARS-CoV-2 wild-type mNeonGreen (WT-mNG) in cell culture. Briefly, Vero E6 (African green monkey kidney) and Calu-3 2b4 (human respiratory) cells were infected at a low multiplicity of infection (MOI) of 0.01 with recombinant SARS-CoV-2 WT-mNG or the KR, R203M, or T205I mutants. Viral titer was then monitored for 48 hours post-infection (hpi). In Vero E6 cells, while the KR and R203M mutants grew to higher titers at 24 hpi, all viruses grew to equivalent titers by 48 hpi (Fig. 1C). The T205I mutation had no significant differences in Vero E6 cells. By contrast, at 24 hpi, all three mutants grew to a higher titer than WT-mNG in Calu-3 2b4 cells (Fig. 1D). At 48 hpi, the KR and R203M mutants, but not the T205I mutant, maintained significantly higher titers than WT-mNG. These data indicate that the KR and R203M mutations uniformly enhance SARS-CoV-2 replication, while the T205I mutation only increases titer in human respiratory cells.

### SR mutants enhance fitness relative to wildtype SARS-CoV-2

**Fig 2:**
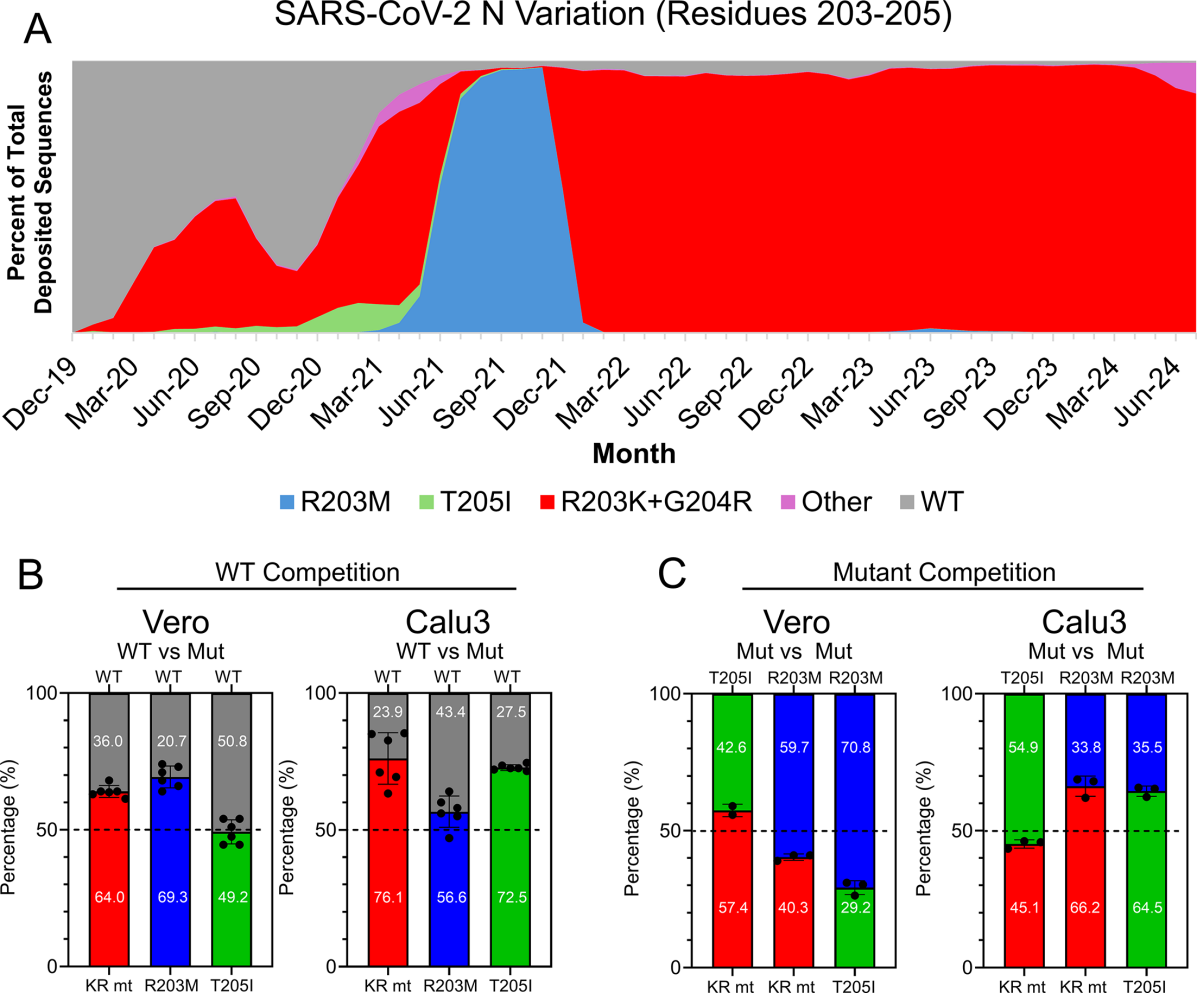
Relative fitness of the KR, R203M, and T205I mutants by direct competition assay. (**A**) Historical variation in SARS-CoV-2 nucleocapsid residues 203–205. Shown are the amino acid frequencies for nucleocapsid residues 203–205 reported to the GISIAD. Raw sequences were collected, enumerated by month of collection, and graphed as a rolling percentage of total sequences reported during that month. (**B, C**) Direct competition assays were performed by infecting Vero E6 and Calu-3 2b4 cells at a 1:1 ratio with the indicated viruses and harvesting cellular RNA at 24 hpi. In (**B**), SARS-CoV WT-mNG was competed against each mutant as indicated. For (**C**), the KR, R203M, and T205I mutants were competed against each other. Bars indicate the mean percentages of cellular RNA, and individual replicates are displayed as points. Error bars indicate ± s.d., (*n* ≥ 3).

The relative prevalence of the KR, R203M, and T205I mutations tracked closely with the prevalence of their respective variants, increasing and decreasing in frequency with the emergence and decline of each VOC (Fig. 2A). Based on this observation, we hypothesized that fitness differences between the KR, R203M, and T205I mutants contributed to the displacement of the original WT strain and one VOC by another. Therefore, we assessed the fitness of each SR mutant relative to SARS-CoV-2 WT-mNG and each other using direct competition assays, which offer increased sensitivity to determine fitness advantages (13). We first competed each SR mutant against SARS-CoV-2 WT-mNG by infecting Vero E6 and Calu-3 2b4 cells with an equal ratio of SR mutant to WT-mNG. At 24 hpi, whole-cell RNA was extracted, and the ratio of mutant to WT-mNG viral RNA was determined by next-generation sequencing (14). In Vero E6 cells, the KR and R203M mutants outcompeted WT-mNG, while the T205I mutant exhibited almost identical fitness compared to WT-mNG (Fig. 2B, left panel). In Calu-3 2b4 cells, the KR and T205I mutants strongly outcompeted WT-mNG (Fig. 2B, right panel), mirroring the increased viral titer exhibited by these mutants (Fig. 1D). By contrast, despite growing to a similar titer as the KR and T205I mutants, the SARS-CoV-2 R203M mutant had only a modest fitness advantage over WT-mNG during direct competition (Fig. 2B, right panel). Together, these data indicate that while SR mutants have greater fitness than the original WT SARS-CoV-2, the extent of this advantage depends on cell type and the specific SR mutation. These data also suggest that the R203M mutation exhibits a smaller fitness advantage against wild type under conditions of direct competition, a finding not predicted by classic replication assays.

### Fitness differences between SR mutants are cell type specific

To evaluate whether N mutations at positions 203–205 contribute to VOC replacement events, we assessed the relative fitness of SARS-CoV-2 harboring each SR mutation during direct competition against each other. Vero E6 or Calu-3 2b4 cells were infected with two different SR mutants at an equal ratio, with assays performed to assess all possible combinations (KR vs T205I, KR vs R203M, T205I vs R203M). In Vero E6 cells, the R203M mutant exhibited the highest fitness, outcompeting both the KR and T205I mutants, while the KR mutant had a small advantage over the T205I mutant (Fig. 2C, left panel). By contrast, in Calu-3 2b4 cells, studies revealed that the T205I mutant outcompeted both the KR and R203M mutants, while the KR mutant outcompeted the R203M mutant (Fig. 2C, right panel). Taken together, these data suggest the relative fitness between different SR mutations is dependent on cell type. However, it also suggests that in human respiratory cells, the T205I mutation exhibits the highest fitness.

### The KR mutation alone confers partial resistance to the interferon response

A notable feature of Vero cells is their inability to induce a type I interferon (IFN) response following viral infection. Since the CoV N protein is known to inhibit IFN signaling, one potential explanation for the differential impact of SR mutations on SARS-CoV-2 fitness during the infection of Vero E6 and Calu-3 2b4 cells is that these mutations modulate the IFN response. To test this, we first measured the induction of IFNβ and the ISGs IFIT1 and IFIT3 in Calu-3 2b4 cells. Briefly, Calu-3 2b4 cells were infected at an MOI of 1.0 with SARS-CoV-2 WT-mNG or the KR, R203M, or T205I mutants. At 24 hpi, whole-cell RNA was harvested and analyzed by RT-qPCR. No significant differences were observed in the expression of these genes among the different viruses (Fig. S1A), suggesting that the SR mutations do not inhibit the induction of the IFN response.

To confirm these findings, we also assessed whether SR mutations could affect SARS-CoV-2 sensitivity to IFN pre-treatment. Vero E6 cells were pre-treated with 1,000 units of recombinant IFNα for 18 hours and then infected at an MOI of 0.01 and viral titers were measured at 48 hpi. While the WT-mNG, R203M, and T205I mutants showed comparable reductions in titers following IFNα treatment, the KR mutant maintained significantly higher titers, indicating partial resistance to IFNα (Fig. S1B). These findings suggest that, although the SR mutations do not affect the induction of the type I IFN response, the KR mutation may provide partial resistance to its antiviral effects.

### The R203M and T205I mutations enhance *in vivo* infection

**Fig 3:**
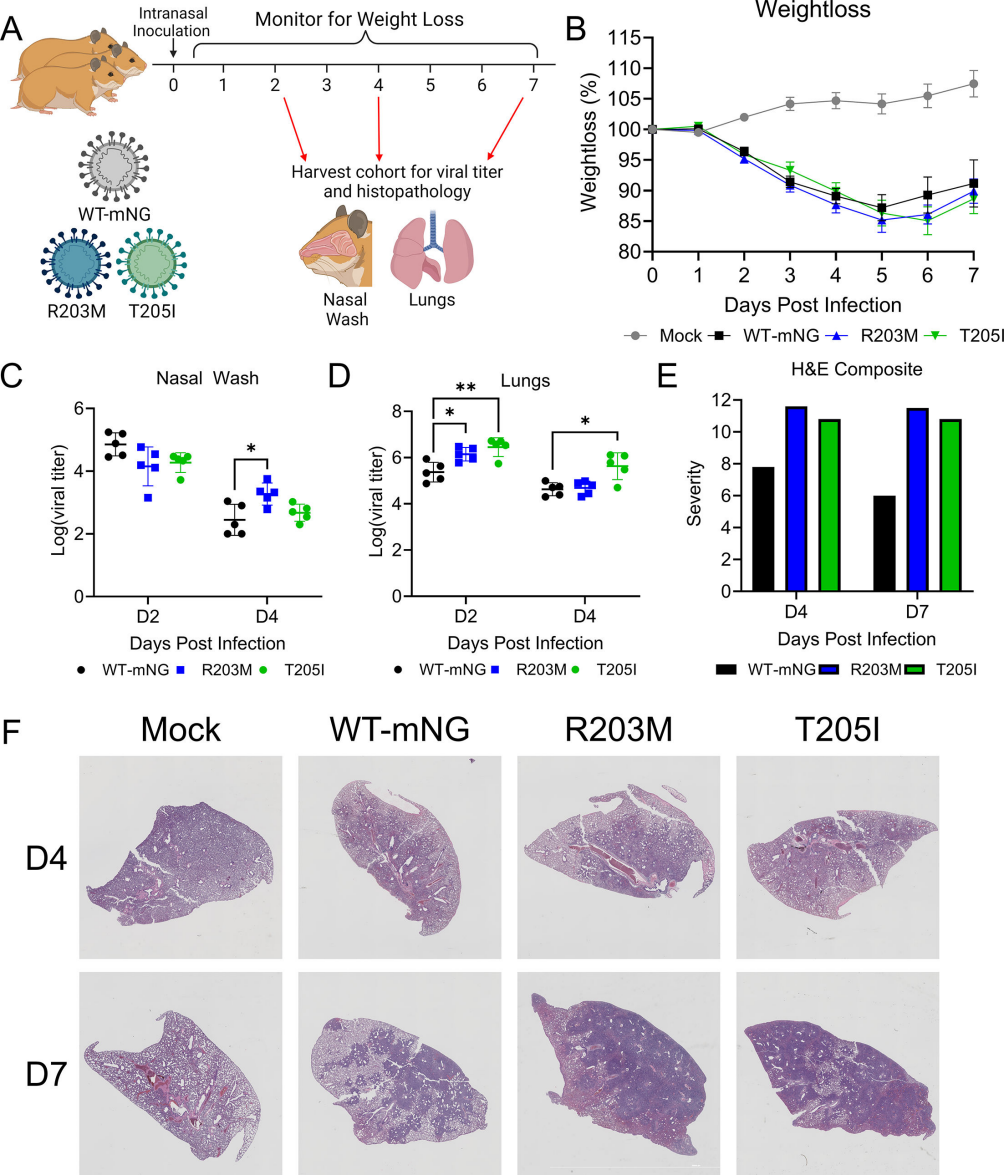
The R203M and T205I mutants increase lung replication and pathogenesis. (**A**) Schematic of infection of hamsters with SARS-CoV-2. (**B through F**) Three-to-four-week-old male hamsters were intranasally infected with PBS alone (mock - gray) or 10^4^ FFU of SARS-CoV-2 WT-mNG (black), R203M (blue), or T205I (green). (**B**) Mean percent change in weight ±S.E.M (*n* ≥ 10). (**C, D**) Viral titer in the nasal washes (**C**) and lungs (**D**). Points indicate the values of individual animals, while horizontal lines represent means and error bars ± s.d. (E, F) Hematoxylin and eosin (H&E) staining was performed on fixed left lung sections. A blinded clinical pathologist then rank ordered all slides based on the severity of lesions, and a composite score was generated by averaging the rank order for each group after unblinding (*n* ≥ 4 for each group) (**E**). Representative images from H&E staining are shown in (**F**). For all experiments, statistical significance was determined by one-way ANOVA followed by Tukey’s multiple comparison test. (*) *P* ≤ 0.05; (**) *P* ≤ 0.01.

Having previously established that the KR mutation enhances SARS-CoV-2 pathogenesis in golden Syrian hamsters (3), we next investigated whether the R203M and T205I mutations exhibit similar effects. Golden Syrian hamsters were intranasally inoculated with PBS (mock) or 10^4^ focus-forming units (FFU) of SARS-CoV-2 WT-mNG, the R203M mutant, or the T205I mutant. Animals were then monitored for weight loss and disease progression for 7 days post-infection (dpi). On 2, 4, and 7 dpi, cohorts of animals were euthanized for the collection of nasal washes and lung tissue (Fig. 3A). Interestingly, unlike the KR mutant (3), neither the R203M nor the T205I mutation exhibited significant differences in weight loss relative to SARS-CoV-2 WT-mNG (Fig. 3B). Despite this, at day 4, hamsters infected with SARS-CoV-2 R203M exhibited higher titers in the nasal passages compared to those infected with WT-mNG (Fig. 3C). In addition, animals infected with the R203M or T205I mutants had increased lung titers compared to those infected with WT-mNG at 2 dpi, while T205I alone had increased lung titer at 4 dpi (Fig. 3D). Together, these data suggest that despite no differences in weight loss, the R203M and T205I mutations increase SARS-CoV-2 replication *in vivo*.

To further examine disease, we performed hematoxylin and eosin (H&E) staining on lung tissue from infected animals at 4 and 7 dpi. These sections were subsequently evaluated by a board-certified pathologist in a blinded manner (Fig. 3E). Animals infected with SARS-CoV-2 WT-mNG, the R203M mutant, and the T205I mutant all exhibited significant inflammation and infiltration including arterial margination of mononuclear cells, interstitial pneumonia, perivasculitis, and alveolar hemorrhaging (Fig. 3F). While absent in the mock-infected animals, disease and pathology were more severe in SARS-CoV-2-infected animals as the infection progressed from day 4 to day 7. Importantly, pathology was more severe and widespread in the R203M and T205I mutants compared to those infected with WT-mNG, as determined by composite scoring (Fig. 3E). Together, these data suggest that despite having minimal effect on weight loss, the R203M and T205I mutations enhance SARS-CoV-2 lung pathology relative to control SARS-CoV-2 infection.

**Fig 4:**
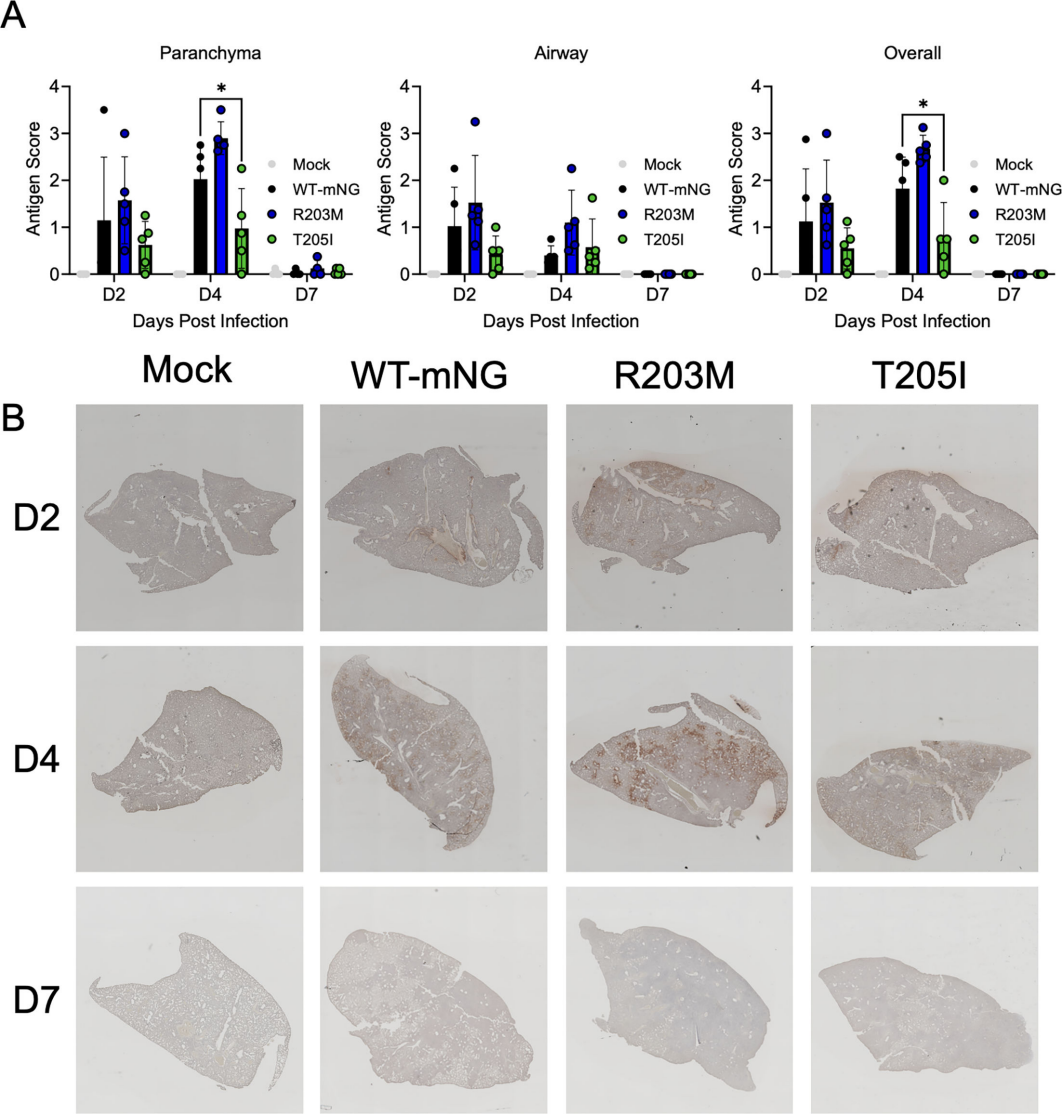
Lung antigen staining from R203M- and T205I-infected hamsters. (**A, B**) Lung sections from Mock or SARS-CoV-2 WT-mNG, R203M, and T205I-infected hamsters were antigen stained for SARS-CoV-2 N. (**A**) Blinded staining scores for the parenchyma (left), airways (middle), and overall staining (right). Bar graphs indicate mean score ± s.d. with scores for individual animals represented as dots. Statistical significance was determined by one-way ANOVA followed by Tukey’s multiple comparison test. (*) *P* ≤ 0.05. (**B**) Representative images from antigen staining experiments.

Given the increases in lung titers and disease found in R203M- and T205I-infected animals, we next evaluated SARS-CoV-2 antigen staining to assess infection in the lung. Using immunohistochemistry staining (IHC) and an antibody targeting SARS-CoV-2 nucleocapsid, we found that the antigen was present in the airways and parenchyma for all infection groups at day 2. By day 4, viral antigen was expanded in the parenchyma in WT, R203M, and T205I-infected lungs. By day 7, minimal viral antigen staining was observed, consistent with clearance of the virus at late times. Importantly, while no significant differences in antigen scoring were observed, the R203M mutant staining trended higher and was more intense than WT-mNG (Fig. 4A and B). Interestingly, despite growing to higher titer *in vivo*, the T205I mutant showed reduced distribution of viral antigen both overall and within the lung parenchyma. One possible explanation is that infection with the T205I mutant may be more focal, achieving high titers through concentrated replication in localized areas. Alternatively, this mutation may shift infection toward the large airways versus the parenchyma. Since airway epithelial cells are more prone to sloughing, they may release more virus into the airways, whereas parenchymal infection may support greater cell-to-cell spread but reduce extracellular viral release. Another possibility is that the T205I mutation alters N protein expression, trafficking, or stability, resulting in lower levels of N antigen detectable by IHC. Despite the apparent discordance between antigen staining and viral titers, our overall findings demonstrate that both the R203M and T205I mutants enhance viral replication and disease severity relative to wild-type SARS-CoV-2.

### Different SR mutations confer unique patterns of nucleocapsid phosphorylation

**Fig 5:**
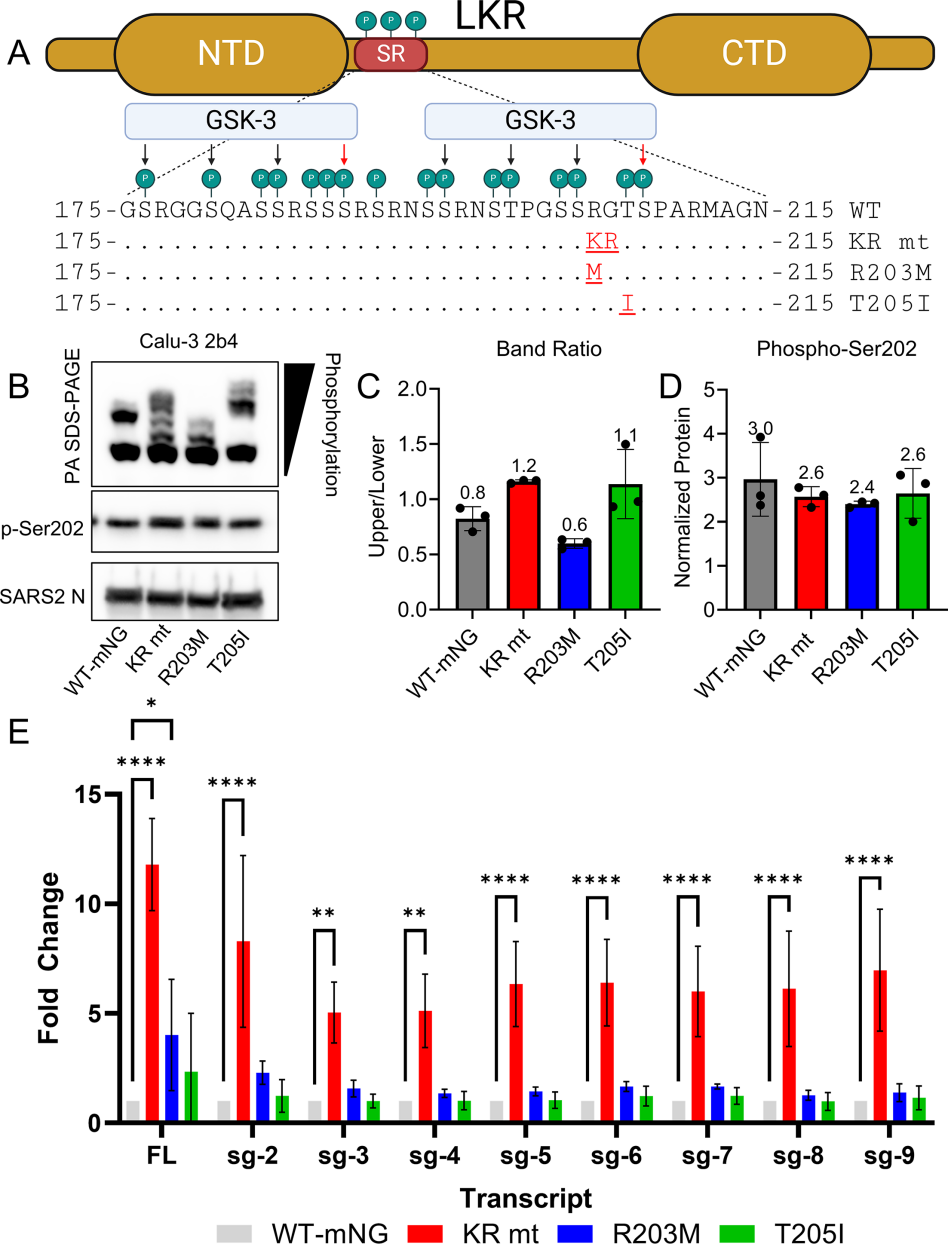
The KR, R203M, and T205I mutations differentially modulate the phosphorylation of SARS-CoV-2 N. (**A**) Schematic of the phosphorylation of SARS-CoV-2 N’s serine-arginine (SR) domain by GSK-3. Priming residues are indicated by red arrows, and additional GSK-3-dependent phosphorylation sites are shown in black arrows. (**B through D**). Whole-cell lysates were harvested from Calu-3 2b4 cells infected with SARS-CoV-2 WT-mNG or the KR, R203M, or T205I mutants at MOI 0.01 (*n* = 3). (**B**) 48 hpi, whole-cell lysates were taken and analyzed by PA SDS-PAGE and standard western blotting. (**C**) Densitometry was performed on PA SDS-PAGE blots, and the ratio of the lowest bands to all other bands was calculated. (**D**) Densitometry was performed on p-Ser202 blots and normalized to loading. Bars represent the mean signal for each group and error bars ± s.d., and individual replicates are depicted as points (*n* = 3). (**E**) Calu-3 2b4 cells were infected with SARS-CoV-2 WT-mNG or the KR, R203M, or T205I mutants at an MOI of 1.0 (*n* = 3). Levels of full-length (FL) and each sub-genomic (sg) transcript were then determined by RT-qPCR. For densitometry studies, significance was analyzed by one-way ANOVA. For sub-genomic transcript levels, significance was determined by two-way ANOVA with (*) *P* ≤ 0.05, (**) *P* ≤ 0.01, and (****) *P* ≤ 0.0001.

The SR domain is the primary site of phosphorylation of the SARS-CoV-2 N protein (Fig. 5A). We previously reported that the KR mutation was sufficient to augment the phosphorylation of SARS-CoV-2 N (3). Thus, we hypothesized that the R203M and T205I mutations would elicit similar effects. To explore this question, we used phosphate-affinity (PA) SDS-PAGE, which separates different phospho-species of a protein using a Zn^2+^ compound called Phos-tag, which selectively binds to phosphorylated amino acids (15). When added to an acrylamide gel, the electrophoretic mobility of proteins decreases proportionally with the number of phosphorylated residues. This technique also allows different phospho-species of the same protein to be separated, creating a laddering effect, with each band representing a distinct phosphorylation state. When combined with western blotting, PA SDS-PAGE enables the analysis of a protein’s phosphorylation state without the use of phospho-specific antibodies.

To investigate the effects of SR mutations on SARS-CoV-2 N phosphorylation, Calu-3 2b4 cells were infected with SARS-CoV-2 WT-mNG or the KR, R203M, or T205I mutant viruses at an MOI of 0.01. After 48 hpi, whole-cell lysates were harvested, and SARS-CoV-2 N phosphorylation was examined by PA SDS-PAGE followed by western blotting. Consistent with our previous report (3), cells infected with WT-mNG exhibited a two-band pattern, while the KR mutant exhibited a laddered pattern consisting of bands of increased, intermediate, and low mobility relative to WT-mNG (Fig. 5B). By contrast, the R203M mutation exhibited a three-band pattern, with a single dark lower band and two fainter intermediate bands, but no low mobility/highly phosphorylated bands (Fig. 5B). Finally, the T205I mutant exhibited its own 4-band pattern, consisting only of a dark lower band and three slow-moving/highly phosphorylated bands (Fig. 5B). Together, these data indicate that each SR mutation uniquely affects the SARS-CoV-2 N phosphorylation state.

Having demonstrated that each SR mutation induces qualitative differences in the pattern of phosphorylation, we next determined whether these changes altered the percentage of SARS-CoV-2 N that becomes phosphorylated. Two methods were used for this analysis. First, we performed densitometry on our PA SDS-PAGE blots to determine the relative ratios of the least/unphosphorylated band to all other bands (Fig. 5C). Despite drastic qualitative differences, none of the SR mutations resulted in statistically significant changes in the phosphorylation ratio. To confirm these findings, we also performed standard western blotting using a phospho-specific antibody targeting SARS-CoV-2 N serine 202 (Fig. 5B, middle panel). Consistent with PA SDS-PAGE, densitometric analysis revealed that the total level of phosphorylation at serine 202 was not affected by any of the SR mutations (Fig. 5D). Together, these data suggest that while SR mutations do not affect the percentage of SARS-CoV-2 N that is phosphorylated within the cell, they do affect the pattern of phosphorylation, producing intermediately and/or highly phosphorylated SARS-CoV-2 N proteins depending on the specific mutation.

### The KR mutation alone enhances the synthesis of viral RNA

The CoV nucleocapsid protein is known to play a role in the replication and transcription of viral RNA (16, 17). Having previously shown that the KR mutation enhances viral RNA synthesis (3), we sought to determine whether the R203M and T205I mutations produce similar effects. To test this, Calu-3 2b4 cells were infected at an MOI of 1.0 with SARS-CoV-2 WT-mNG or one of the SR mutants. At 24 hpi, whole-cell RNA was harvested, and the levels of full-length (FL) and subgenomic (sg) RNAs were analyzed by RT-qPCR. Consistent with our previous study, the KR mutant significantly increased the levels of all SARS-CoV-2 transcripts relative to WT-mNG (Fig. 5E). By contrast, the R203M mutant produced only a modest increase in FL RNA, and neither the R203M nor T205I mutants significantly elevated sgRNA levels compared to WT-mNG. These findings suggest that among the SR mutations found in SARS-CoV-2 VOCs, only the KR mutation significantly impacts viral RNA synthesis.

### Enhanced replication of SR mutants is lost during infection of bat cells

**Fig 6:**
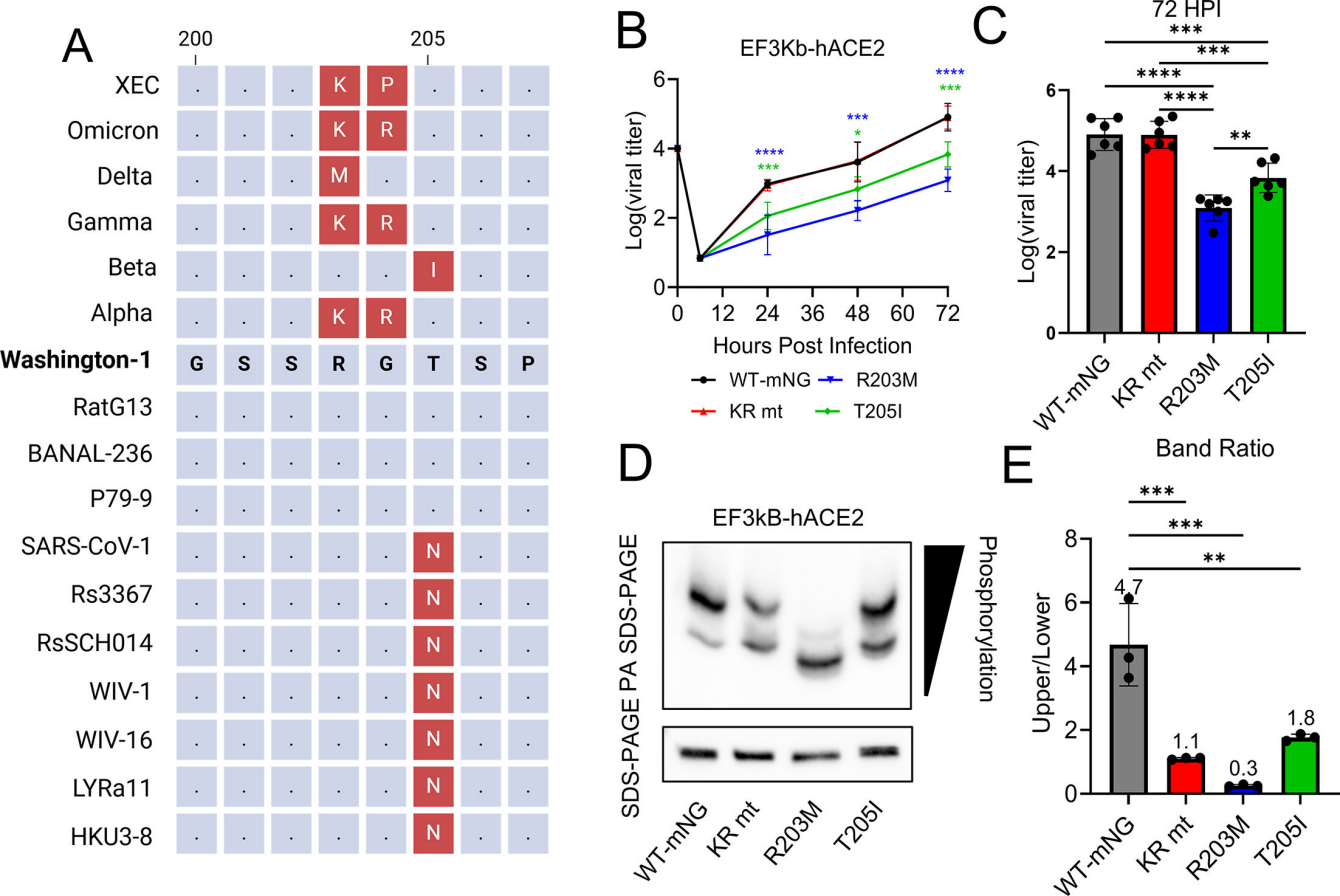
Species-specific differences in SARS-CoV-2 N phosphorylation correlate with SR mutants’ impact on replication. (**A**) Schematic depicting the genetic variation in the SARS-CoV-2 N’s highly variable region compared with other Sarbecoviruses. Differences from SARS-CoV-2 WA-1 are shown in red. (**B through E**) Big brown bat-derived Efk3B-hACE2 cells were infected at an MOI of 0.01 with SARS-CoV-2 WT-mNG or the KR, R203M, or T205I mutants. Viral titer was then determined in sampled supernatants, with the full-time course shown as a line graph (**B**) and endpoint titers as a bar graph (**C**); (*n* = 6). Whole-cell lysates were then taken, and PA SDS-PAGE and standard western blot analysis performed (**D**); (*n* = 3). Densitometry was performed on PA SDS-PAGE blots comparing the ratio of the upper to the lower band (**E**). Line graph depicts mean viral titer ±s.d. For bar graphs, bars represent means, error bars are ± s.d., and individual replicates are depicted as points. Statistical significance was determined by one-way ANOVA followed by Tukey’s multiple comparison test. (*) *P* ≤ 0.05; (**) *P* ≤ 0.01; (***) *P* ≤ 0.001; (****) *P* ≤ 0.0001.

While residues 203–205 are variable among SARS-CoV-2 VOC, the corresponding region in other Sarbecoviruses is highly conserved (Fig. 6A). This fact suggests that each SARS-CoV-2 VOC acquired its specific SR mutation due to convergent evolution and positive selection for human infection. To test this hypothesis, we examined infection in bat-derived cells to mimic infection in the zoonotic reservoir of Sarbecoviruses. Efk3B-hACE2 cells are a kidney cell line isolated from *Eptesicus fuscus* (big brown bat). While permissive to some CoVs, stable expression of human ACE2 is required for SARS-CoV-2 infection (12). Efk3B-hACE2 cells were infected at an MOI of 0.01 with SARS-CoV-2 WT-mNG or the KR, R203M, and T205I mutants, and viral titers were monitored for 72 hpi. Strikingly, the increased replication conferred by SR mutations in Vero E6 and Calu-3 2b4 cells was absent during infection of Efk3B-hACE2 cells (Fig. 6B and C). Both the R203M and T205I mutants grew to significantly lower titers than WT-mNG at all time points, indicating that these mutations reduce replication in bat cells (Fig. 6B and C). By contrast, the KR mutant grew to titers equal to WT-mNG, indicating that while the KR mutation is not deleterious for infection of bat cells, the KR mutant lacks the replication advantage seen in Vero E6 and Calu-3 2b4 cells. Together, these data support the hypothesis that the SR mutations that emerged in SARS-CoV-2 VOC are specific adaptations for human infection and suggest species-specific mutations occur in CoV N proteins to facilitate host range expansion.

Having established that SR mutations have reduced replication in Efk3B-hACE2 cells, we next determined whether SARS-CoV-2 N phosphorylation was similarly affected. We began by examining whole-cell lysates from infected Efk3B-hACE2 cells using PA-SDS-PAGE (Fig. 6D). WT-mNG-infected cells exhibited a two-band pattern similar to what was seen during infection of Calu-3 2b4 cells (Fig. 6D). However, the KR and T205I mutations also exhibited the same two-band pattern with equal electrophoretic mobility (Fig. 6D), contrasting sharply with the pattern seen during infection of Calu-3 2b4 cells (Fig. 5B). In contrast to the other three viruses, R203M-infected cells showed a pattern consisting of only one prominent and one very faint band, each with increased mobility/reduced phosphorylation relative to the other viruses (Fig. 6D). Analyzing these bands by densitometry and calculating the ratios of the upper and lower bands, we found that all three SR mutants had significantly decreased SARS-CoV-2 N phosphorylation relative to WT-mNG (Fig. 6E). Together, these data suggest that SR mutations decrease SARS-CoV-2 N phosphorylation during infection of bat cells, providing a molecular explanation for the lack of a replication advantage.

## DISCUSSION

This manuscript presents a comparative analysis of three mutations in the SARS-CoV-2 nucleocapsid (N) protein present among Variants of Concern (VOC). Collectively termed the “SR” mutants due to their location in the SARS-CoV-2 N protein’s serine-arginine (SR) domain, these mutations include R203K + G204R (KR), R203M, and T205I. Using our reverse genetic system (11), the T205I mutation was introduced into the Washington-1 (WA-1) background and compared to the KR and R203M mutants generated previously with this system (3). We demonstrate that all three SR mutations enhance replication and fitness relative to wild-type SARS-CoV-2 *in vitro*. By contrast, when competed against each other, the relative fitness of SR mutants was found to be cell line specific. In Vero E6 cells (African green monkey kidney), the R203M mutation exhibited the greatest fitness, while in Calu-3 2b4 cells (human respiratory), the T205I mutation outcompeted the others. During *in vivo* infection of golden Syrian hamsters, the R203M and T205I mutants enhanced viral replication and lung pathology without significantly affecting weight loss. These findings differ from the KR mutation, which increased weight loss, pathology, and viral RNA in the lung, but had no significant impact on viral titer in our hands (3). Interestingly, each SR mutant induced a unique pattern of SARS-CoV-2 N phosphorylation, differing from both wild-type and each other. Interestingly, while all three mutants increase viral titer, only the KR mutant enhances the replication/transcription of viral RNA. The unique patterns of SARS-CoV-2 N phosphorylation provide a molecular explanation for why these three mutations induce similar, but not identical, phenotypes *in vitro* and *in vivo*. Using cells derived from *Eptesicus fuscus* (big brown bats) expressing human ACE2 to mimic infection in SARS-CoV-2′s zoonotic reservoir (12), we showed that the R203M and T205I mutations all reduced replication and SARS-CoV-2 N phosphorylation relative to wildtype virus, while the KR mutant grew to equal titer. Collectively, these findings suggest that the emergence of three distinct mutations at residues 203–205 of SARS-CoV-2 N in VOC is the result of convergent evolution and positive selection for human infection.

The SR mutations are located at the C-terminus of SARS-CoV-2 N’s SR domain, which is the primary site of its phosphorylation (18–21). According to a model proposed by Yaron et al., the phosphorylation of SARS-CoV-2 N is initiated by SRPK, which adds two initial “priming” phosphate groups on serine residues 188 and 206 (Fig. 5A) (18). GSK-3 then binds these primed sites, adding three additional phosphate groups every four amino acids upstream (18). Interestingly, the KR, R203M, and T205I mutations are sandwiched between the priming serine 206 and the GSK-3 targeted serine 202 (Fig. 5A). This suggests that SR mutants may function to optimize SARS-CoV-2 N phosphorylation by human kinases. Supporting this idea is our finding that while SR mutations enhance N phosphorylation in human cells, they reduce N phosphorylation in bat cells (Fig. 5B and C and Fig. 6D and E). By contrast, the observation that phosphorylation at serine 202 is not affected by SR mutations complicates such a straightforward explanation (Fig. 5B, middle panel, and D). Instead, each SR mutation may selectively influence GSK-3-mediated phosphorylation at specific downstream residues, such as threonine 198 or serine 194 (Fig. 5A). Alternatively, these mutations could alter phosphorylation by modulating the activity of casein kinase 1 (CK1), which targets residues 193, 197, 201, and 205 following priming by GSK-3 (18). Given that all three SR mutants exhibit distinct phosphorylation patterns, yet only the KR mutant significantly enhances viral RNA synthesis, identifying the specific residues differentially phosphorylated by each mutation could provide mechanistic insights into how SARS2 N phosphorylation governs viral RNA synthesis.

While residues 203–205 are highly variable in SARS-CoV-2, these amino acids are relatively conserved among other Sarbecoviruses (Fig. 6A). A notable exception to this rule is the early pandemic SARS-CoV-2 strains and SARS2-like bat-CoVs (e.g., BANAL viruses), which encode a threonine at residue 205, while SARS-CoV-1 and other Sarbecoviruses encode an asparagine. The importance of this asparagine-threonine substitution is unclear. For instance, both serines and threonines can be phosphorylated, while asparagine cannot. It is possible that the addition of a phosphate group at threonine 205 may interfere with the phosphorylation of serine 206, leading to mis-priming and inhibition of GSK-3-dependent phosphorylation upstream. If true, this would suggest that SR mutations found in VOC arose to correct this mis-priming. Further studies are needed to investigate the importance of this asparagine-to-threonine substitution between the SARS-CoV-2 and SARS-CoV-1 N proteins.

Another unanswered question is why the KR mutation, and not one of the other SR mutations, came to dominate in nearly all extant SARS-CoV-2 lineages (Fig. 2A). Our fitness data from Calu-3 2b4 cells suggest that the T205I mutation exhibits the highest fitness in human respiratory cells (Fig. 2C). These data are discordant with historical sequencing data from the GISAID database, showing the Beta (T205I) and Alpha (KR) variants being displaced by Delta (R203M) between May and December 2021 (Fig. 2A). While Delta was eventually displaced by Omicron, harboring the more fit KR genotype, neither the initial emergence of the R203M mutation nor the disappearance of the T205I mutation would be predicted by our competition studies. Two possible explanations exist. In one scenario, the fitness differences between SR mutations may be small enough that mutations elsewhere in the genome become the deciding factor during inter-variant competition. Supporting this view are published reports describing how a mutation in the Spike (S) residue 681 contributed to Delta’s displacement of Alpha (22). Alternatively, the R203M mutation may impact fitness and/or transmission in a manner not modeled by our *in vitro* competition experiments. For instance, R203M, but not other SR mutants, enhanced viral titer in the nasal washes of infected hamsters (Fig. 3C). If the R203M mutation alone enhances infection of the upper respiratory system and thereby augments transmission, this could help explain its rapid displacement of variants harboring the KR and T205I mutations. In addition to these historical VOC replacement events, it should be noted that the emerging XEC variant harbors a unique R203K, G204P mutation (Fig. 6A). How this novel “KP” mutation augments replication, fitness, and pathogenesis needs to be explored.

It is also necessary to contextualize our results with other studies examining SR mutants. While there is a consensus that both the KR and R203M mutations enhance fitness, there are competing views regarding the mechanism. For instance, two studies have demonstrated that a novel transcriptional regulatory sequence (TRS) is generated by the KR mutation, leading to the expression of a truncated version of SARS-CoV-2 N referred to as N* or N.iORF3 (6, 7). Comparing recombinant mutants, a SARS-CoV-2 KR mutant harboring the TRS sequence exhibited altered replication compared to a SARS-CoV-2 KR mutant where codon usage was altered to maintain protein sequence while eliminating the novel TRS site (7). This suggests that this novel TRS site contributes to the enhanced fitness of the KR mutation. However, neither the R203M nor T205I mutations create a novel TRS site, suggesting that the expression of a truncated N protein does not account for the convergent evolution in the highly variable 203–205 regions. Alternatively, data from Syed et al. using virus-like particle (VLP)-based reporter assays suggest that mutations in the SR domain, and the R203M mutation in particular, enhance the packaging of viral RNA (4). However, contrasting our data with genuine virus, Syed et al.’s data suggest that neither the G204R nor T205I mutations enhance packaging. Thus, once again, enhanced packaging of viral RNA cannot account for the independent emergence of three mutations within the SR domain’s highly variable motif. In a third and final view, Wu et al. show that while the KR mutation enhances viral titer in cell culture supernatants, extracellular viral RNA levels are not affected (5). These data suggest that the KR mutation may enhance infectivity within the virion itself by an unknown mechanism. How these three potential mechanisms relate to the enhanced SARS-CoV-2 N phosphorylation reported in this manuscript needs to be further explored.

There are several limitations of this study that warrant consideration. First, although we examined the effects of individual SR mutations in isolation, circulating VOCs often contain multiple mutations within the SARS2 N protein, which may interact synergistically. The approach taken here allows us to isolate and characterize the functional impact of each SR mutation individually; however, potential epistatic interactions with other co-evolving mutations are not captured. Second, all mutations were introduced into an attenuated SARS-CoV-2 reporter strain in which ORF7 has been replaced by mNeonGreen (3, 23). This attenuation may mask or modulate the full pathogenic potential of the SR mutations, particularly *in vivo*. Finally, while EF3kB-hACE2 cells provide a useful platform to evaluate infectivity in bats, these cells originate from kidney tissue, whereas zoonotic CoVs are generally believed to infect the bat gastrointestinal tract. Therefore, the reduced replication and phosphorylation reported in this study may reflect tissue-specific rather than species-specific differences.

Overall, our work establishes that variant mutations found in the SR domain of Nucleocapsid augment SARS-CoV-2 infection. In addition, each of these mutations induces a unique pattern of SARS-CoV-2 N phosphorylation, providing a molecular explanation for their similar but non-identical effects on SARS-CoV-2 infection. Interestingly, we find that the capacity of these mutations to enhance SARS-CoV-2 infection is reversed when infecting bat cells. These data suggest that the KR, R203M, and T205I mutations are specific adaptations for human infection, adapting SARS-CoV-2 N for phosphorylation by human kinases. These findings underline the importance of mutations outside of the spike protein for SARS-CoV-2′s emergence and adaptation for human infection and pathogenesis.

## MATERIALS AND METHODS

### Generation of recombinant virus

The sequence of the wild-type (WT) SARS-CoV-2 is based on the USA-WA1/2020 strain, originally isolated by the USA Centers for Disease Control and Prevention (24). Recombinant viruses were generated using cDNA clones as previously described (11). The construction of all SARS-CoV-2 viruses was approved by the University of Texas Medical Branch Institutional Biosafety Committee.

### Cell culture

Vero E6 cells were grown in a media cocktail of DMEM (Gibco #11965-092), 10% fetal bovine serum (Cytiva #SH30071.03), and 1% antibiotic/antimycotic (Gibco #5240062). Calu-3 2b4 cells were grown in DMEM with 10% defined fetal bovine serum (Cytiva #SH30070.03), 1% antibiotic/antimycotic, and 1 mg/mL sodium pyruvate. EFk3B-hACE2 cells were derived from the existing EFk3b cell line, a kidney epithelial cell line from the big brown bat (Eptesicus fuscus) that was previously immortalized using SV40 large T-antigen (12). To generate an ACE2-expressing derivative cell line, EFk3B cells were transfected with a plasmid encoding human ACE2. Forty-eight hours post-transfection, a single-cell suspension was generated with trypsin, stained with a human ACE2-specific antibody, and then sorted via Fluorescence-Activated Cell Sorting (FACS) on a BD Aria Fusion instrument to isolate ACE2-positive cells. The resulting EFk3B-hACE2 cell population was cultured in DMEM supplemented with 10% fetal bovine serum and 1% antibiotic/antimycotic and cryopreserved. Stable expression of hACE2 was maintained using 1 µg/mL puromycin.

### *In vitro* infections

Cells were infected according to standard protocols described previously (23, 25). Vero E6, Calu-3 2b4, or EFk3B-hACE2 cells were seeded into six-well plates and allowed to grow to confluency. Growth medium was then removed, and cells were infected at a multiplicity of infection of 0.01 or 1.0 with the appropriate virus, as indicated, diluted in PBS. Cells were incubated for 45 minutes at 37°C and 5% CO_2_, after which the inoculum was removed. Cells were then washed three times with PBS, and fresh growth media was added. To sample viral titer, 300 µL (15%) of the supernatant from each well was removed and frozen, while an equal volume of fresh media was added to maintain a consistent volume. All infections were conducted in the Galveston National Laboratory BSL3 facility.

For interferon pretreatment experiments, Vero E6 cells were treated with 1,000 U of recombinant IFNɑ (PBL Assay Science #11200-2) for 18 hours in growth media. Cells were then infected at an MOI of 0.01 as described in the previous section, washed with PBS, and growth media without IFNɑ returned. Cell supernatants were then sampled at 6, 24, and 48 hpi.

### Focus forming assays

To determine viral titer, focus forming assays were performed as previously described (3, 26). Briefly, cell culture supernatants, nasal washes, or homogenized lungs underwent five 10-fold serial dilutions in PBS. Twenty microliter of raw or diluted samples was then incubated for 45 minutes on Vero E6 cells cultured in 96-well plates. Methylcellulose overlay (Sigma #M0512) was then added to each well, and cells were incubated for 24 hours. Methylcellulose was then removed; cells were washed three times with PBS and fixed in 10% formalin for 30 minutes to inactivate SARS-CoV-2. Cells were then permeabilized and stained with a SARS-CoV-1/2 nucleocapsid antibody (Cell Signaling Technology #68344) followed by Alexa-555 anti-mouse (Invitrogen #A28180). Prior to inactivation, all procedures were conducted in the Galveston National Laboratory BSL3 facility.

### Competition studies

Vero E6 or Calu-3 2b4 cells were infected with a 1:1 ratio of two different viruses (WT and mutant, or mutant and mutant, as indicated) as determined by stock titers. Twenty-four hours post-infection, whole-cell RNA was harvested in TRIzol (Invitrogen #15596026) and RNA was extracted with the RNA Miniprep Plus kit (Zymo Research #R2072).

### Next-generation sequencing and data analysis

Total cellular RNAs extracted from infected cells of competition assays were subjected to next-generation sequencing (NGS) to understand the relative ratio of each viral genotype. In addition, RNA derived from virus stocks underwent NGS to verify sequence integrity. In both cases, we used Tiled-ClickSeq (14) to construct NGS libraries, with previously established methods (3). Briefly, RNAs were reverse transcribed with a set of SARS-CoV-2-specific primers and a mixture of azido-NTP/dNTP (1:35 molecular ratio). cDNAs were “click-ligated” to full-length with Illumina i5 adapters and final PCR-amplified with multiplexing barcodes. Gel-selected libraries were sequenced with the Illumina NexSeq 550 platform with pair-end sequencing (120 bp R1 and 30 bp R2).

The raw data were processed with the established bioinformatic pipeline (https://github.com/andrewrouth/TCS), which prepares R1 reads by removing primer sequences (extracted from R2). The remaining R1 reads are mapped to the reference genome (NBCI: NC_045512.2) with bowtie2 (9). Pilon (27) was used to detect genotypic variants and to improve assembly. The relative ratio between viral mutants was calculated based on Pilon-reported mutation rate at each genomic locus.

### Analysis of publicly available genomic data

Available SARS-CoV-2 sequences in the GISAID database were queried on August 14, 2024. Collection dates were binned by month, spanning December 2019 to June 2024. Within each month, the number of sequences with the R203M, the T205I, or the R203K and G204R substitutions or with no substitutions at residues R203, G204, or T205 were quantified directly using the GISAID AA substitutions search feature. The number of sequences with other substitution(s) at one or more of these residues was deduced by subtracting the previously specified sequences from the total number of sequences available in that month.

### Hamster infections

Three- to four-week-old male golden Syrian hamsters were intranasally infected with 100 µL of PBS alone (mock) or 1 × 10^4^ focus forming units (FFU) of SARS-CoV-2 while under isoflurane anesthesia. Animals were monitored for weight loss and clinical disease for 7 days post-infection. At 2, 4, and 7 days post-infection (dpi), animals underwent nasal washing while under anesthesia, followed by euthanasia by CO_2_ asphyxiation and thoracotomy, and lung tissue was harvested. For all experiments, animals were purchased from Envigo. Studies were conducted in accordance with a protocol approved by the UTMB Institutional Animal Care and Use Committee and complied with the United States Department of Agriculture guidelines in a laboratory accredited by the Association for Assessment and Accreditation of Laboratory Animal Care. Procedures involving infectious SARS-CoV-2 were performed in the Galveston National Laboratory ABSL3 facility.

### Histological staining

Left lungs harvested from hamsters were fixed in 10% buffered formalin for 7 days, after which the buffer was exchanged. Fixed tissue was then paraffin-embedded, cut into 5 μM sections, mounted on slides, and stained with hematoxylin and eosin (H&E) by the UTMB Anatomic Pathology Core facility. Alternatively, after embedding on slides, unstained tissue was deparaffinized and antigen-stained in-house with a SARS-CoV-2 N-specific antibody (Sino Biologicals #40143-R001) followed by anti-rabbit secondary antibody (Vector Labs #BA1000). Stained tissues were then developed with ImmPact NovaRed HRP substrate to visualize the antigen (Vector Labs #SK-4805).

### Pathological scoring

H&E slides were scored by a board-certified, blinded pathologist. Microscopic slides of left lung tissue from each hamster were grouped based on the number of days post-intranasal inoculation with SARS-CoV-2. Slides within each group were thoroughly scrambled and examined in a blinded manner to evaluate the severity of pathological lesions. The slides were then rank ordered through serial pairwise comparisons based on lesion severity. Following this, ranks were assigned numerically from 1 (least severe pathology) to the highest number (most severe pathology). After ranking, the identity of each group (mock, wild type, or mutant) was unblinded. The rank scores for each group were summed and then divided by the number of slides in the group to obtain a composite severity score. Some slides were excluded from scoring if abundant polymorphonuclear leukocytes were present, likely indicating superimposed bacterial bronchopneumonia. Separate sums of rank order scores and average severity scores were calculated for each day to generate a composite score.

For SARS-CoV-2 antigen staining, microscopic slides with two sections from the left lung of each hamster were blinded and then independently examined by two researchers. Upon examination, each researcher scored each slide for staining in the parenchyma, airway, and then gave an overall staining score. The scale of possible scores ranged from 0 (no staining) to 4 (severe staining across the entire tissue). Scores from the two researchers were then averaged to generate a single point for each animal.

### Real-time quantitative PCR

SARS-CoV-2 transcript levels were determined as described previously (3). Briefly, RNA from SARS-CoV-2-infected cells was extracted with TRIzol (Invitrogen #15596026) and purified using Direct-zol Miniprep Pluts kits (Zymo Research #R2072) per the manufacturer’s instructions. cDNA was generated using the iScript cDNA Synthesis kit (Bio-Rad #1708891) and RT-qPCR was performed on a CFX Connect Instrument (Bio-Rad #1855200) using Luna Universal qPCR Master Mix (New England Biolabs #M3003). For cellular targets and analysis of sub-genomic RNA ratios, the delta-delta CT method was performed, with 18S ribosomal RNA serving as an internal control.

Primer sequences for cellular targets are as follows: IFNβ (F-AGTAGGCGACACTGTTCGTG; R-AGCCTCCCATTCAATTGCCA), IFIT1 (F- CCAAGGAGACCCCAGAAACC ; R-CGCTACGTGGAGTGAGCT AG), IFIT3 (F-AAGAACAAATCAGCCTGGTCAC; R-TCCCTTGAGACACTGTCTTCC), and 18S (F-CCGGTACAGTGAAACTGCGAATG; R-GTTATCAAGTAGGAGAGGAGCGAG). For analysis of SARS-CoV-2 transcripts, a common forward primer binding upstream of the transcription regulatory sequence (TRS) leader region was used for all transcripts (ACCAACCAACTTTCGATCTCT). For full length and each sub-genomic transcript downstream of the corresponding TRS sequence was used: Full length (CTCGTGTCCTGTCAACGACA), sg2 (TGCAGGGGGTAATTGAGTTCT), sg3 (GCGCGAACAAAATCTGAAGGA), sg4 (AGCAAGAATACCACGAAAGCA), sg5 (ACCGTTGGAATCTGCCATGG), sg6 (GCCAATCCTGTAGCGACTGT), sg7/mNeonGreen (TGCCCTCGTATGTTCCAGAAG), sg8 (ACATTCTTGGTGAAATGCAGCT), and sg9 (CCCACTGCGTTCTCCATTCT).

### SDS-PAGE and western blot analysis

Whole-cell lysates were prepared from SARS-CoV-2-infected Calu-3 2b4 cells at 48 hours post-infection by the addition of cell lysis buffer (Cell Signaling Technology #9803) containing protease (Roche #11836153001) and phosphatase inhibitors (Roche #4906845001). Cell lysis buffer also served as an inactivation reagent. Briefly, cell lysis buffer was added directly to the cell monolayers grown in six-well plates (500 μL/well). Cells were incubated for 5 minutes, and the resulting lysates were collected and clarified by centrifugation. Prior to SDS-PAGE, lysates were mixed with 2× Laemmli buffer (Bio-Rad #1610737) containing 2-mercaptoethanol (Bio-Rad #1610710) and incubated at 95°C for 15 min. For standard SDS-PAGE, 4%–20% Mini-PROTEAN TGX Gels (Bio-Rad #4561093) were used. For Phospho-Affinity SDS-PAGE, 7.5% SuperSep Phos-Tag gels (Wako Chemical #198-17981) were used. After electrophoresis, proteins were transferred to polyvinylidene difluoride (PVDF) membranes. Antibodies used for blotting include SARS-CoV nucleocapsid (gift from Dr. Shinji Makino), SARS-CoV-2 nucleocapsid phospho-serine 202 (generated by Dr. Jacob Nilsson), and anti-Rabbit HRP secondary (Cell Signaling Technology #7074). Images were developed by treating membranes with Clarity ECL substrate (Bio-Rad #1705060) and then imaging on the ChemiDoc MP system (Bio-Rad #12003154).

## Data Availability

All raw sequencing data are available in the NCBI Sequence Read Archive under BioProject number PRJNA1144981. All other data relevant to the present study are shown within the paper

## SUPPLEMENTAL MATERIAL

Figure S1 jvi.02091-24-s0001.docx SR mutants' effects on the type I IFN response to SARS-CoV-2.

ASM does not own the copyrights to Supplemental Material that may be linked to, or accessed through, an article. The authors have granted ASM a non-exclusive, world-wide license to publish the Supplemental Material files. Please contact the corresponding author directly for reuse.
