# Supplementary material for: Convergent evolution in nucleocapsid facilitated SARS-CoV-2 adaptation for human infection
Source: J Virol. 2025 Jun 12;99(7):e02091-24. doi: 10.1128/jvi.02091-24 (PMC12282136; doi:10.1128/jvi.02091-24)
Supplement: Figure S1 — SR mutants' effects on the type I IFN response to SARS-CoV-2. [file jvi.02091-24-s0001.docx]

**Fig. S1: SR mutants’ effects on the Type I IFN response to SARS-CoV-2.** (A) Calu-3 2b4 cells were infected at an MOI of 1.0 with SARS-CoV-2 WT-mNG or the KR, R203M, or T205I mutants. At 48 hpi, whole cell RNA was harvested and the level of IFNβ (left), IFT1 (center), and IFIT3 (right) transcripts were analyzed by RT-qPCR (n=3). Statistical significance was analyzed by one-way ANOVA, followed by Tukey’s multiple comparison test. (B) Vero E6 cells were pre-treated with 1000 U of IFNɑ for 18 hours. Cells were then infected at MOI of 0.01 with SARS-CoV-2 WT-mNG or the KR, R203M, or T205I mutants and viral titer was determined at 48 hpi (n=3). Statistical significance was determined by two-way Anova, followed by Tukey’s multiple comparison test. For all experiments error bars are ± s.d and (**) p≤0.01 and (****) p≤0.0001.


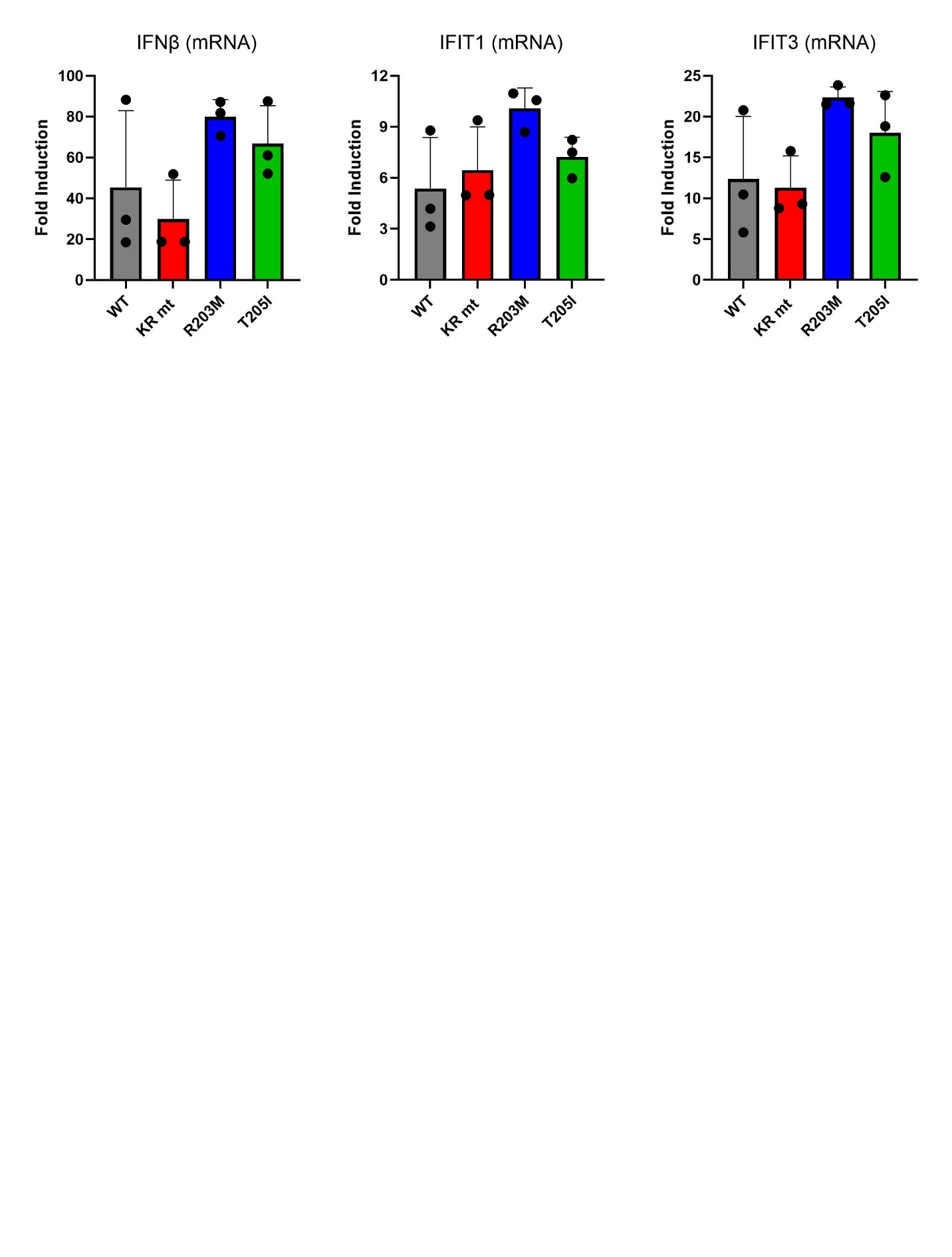


A

B
